# Supplementary material for: Clinical Factors Associated with SFTS Diagnosis and Severity in Cats
Source: Viruses. 2024 May 29;16(6):874. doi: 10.3390/v16060874 (PMC11209305; doi:10.3390/v16060874)
Supplement: Supplementary file 1 [file viruses-16-00874-s001.zip › Figure S1.pdf]

Figure S1. Immunofluorescent analysis of SFTS-NP-Ab detection in viral RNA negative specimens

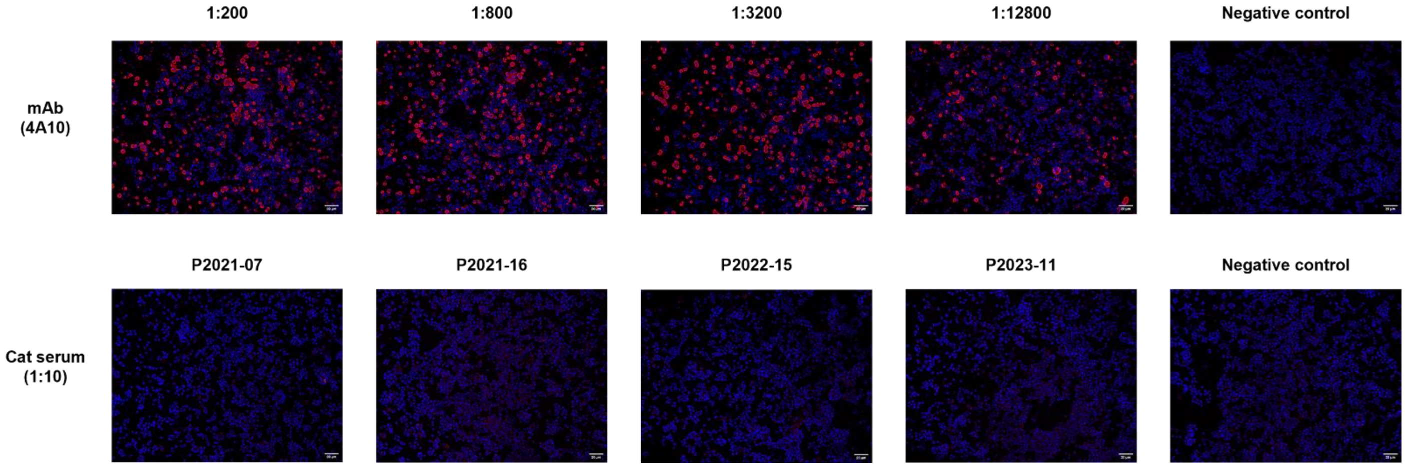

The SFTSV antigen slides were stained with SFTS-NP-mAb (4A10), which was used as a positive control, feline serum samples that were negative for viral RNA, and PBS as a negative control. The secondary antibodies used were Alexa Fluor 594 conjugated donkey anti-mouse and FITC-conjugated goat anti-feline. Nuclei were counterstained with Hoechst stain. Scale bar 20 μm. Partial results of the feline serum are displayed, and the mAb results are shown only for the 1:200, 1:800, 1:3200, and 1:12800 dilutions. Representative images are shown and the cat ID is indicated at the top. FITC, fluorescein isothiocyanate; PBS, phosphate-buffered saline; SFTS-NP-mAb, severe fever with thrombocytopenia syndrome virus monoclonal antibody; SFTSV, severe fever with thrombocytopenia syndrome virus.
