# Supplementary material for: Clinical Factors Associated with SFTS Diagnosis and Severity in Cats
Source: Viruses. 2024 May 29;16(6):874. doi: 10.3390/v16060874 (PMC11209305; doi:10.3390/v16060874)
Supplement: Supplementary file 1 [file viruses-16-00874-s001.zip › Table S1.pdf]

Table S1. Primers used in this study

Primer	Region of gene	Genome position	Sequence
**RT-qPCR**			
SFTS-QPCR-965F	L segment	965-987	5'-GCRAGGAGCAACAARCAAACATC-3'
SFTS-QPCR-1069R	L segment	1039-1059	5'-GCCTGAG TCGGTCTTGATGTC-3'
Primetime® qPCR probe	L segment	1010-1031	FAM/5'-CTCCCRCCCTGGCTACCAAAGC-3'/IBFQ1
**PCR amplify**			
SFTSV-M1-F	M segment	1-20	5'-ACACAGAGACGGCCAACAAT-3'
SFTSV-M1-R	M segment	610-631	5'-TGTCTGGAAATTCACTCTGGCT-3'
SFTSV-M2-F	M segment	296-315	5'-GTGGACTTCTAAGCCCCTGT-3'
SFTSV-M2-R	M segment	2121-2140	5'-TGTCTGGAAATTCACTCTGGCT-3'
SFTSV-M3-F	M segment	1738-1757	5'-GTTTCGTGCAAGCAAGGGAG-3'
SFTSV-M3-R	M segment	3355-3374	5'-AAAGACCGGCCAACACTTCA-3'
**Sequencing**			
SFTSV-M1-R	M segment	610-631	5'-TGTCTGGAAATTCACTCTGGCT-3'
SFTSV-M2-F	M segment	296-315	5'-GTGGACTTCTAAGCCCCTGT-3'
SFTSV-M2-F2	M segment	735-754	5'-CATGAGGGAGCACAAGACCA-3'
SFTSV-M2-F3	M segment	1231-1250	5'-GCAATACACGTCAAAGGGGC-3'
SFTSV-M3-F	M segment	1738-1757	5'-GTTTCGTGCAAGCAAGGGAG-3'
SFTSV-M3-F2	M segment	2265-2284	5'-GAGCGGCATCCCTACTCAAG-3'
SFTSV-M3-F3	M segment	2735-2754	5'-CTACTTGCACAGGGGAGGTG-3'
SFTSV-M3-R	M segment	3355-3374	5'-AAAGACCGGCCAACACTTCA-3'

Table S1 presents the comprehensive details of the primers used in this study. The table includes the following information for each primer: gene region, genome position, and nucleotide sequence of the primers and probes.
