# Supplementary material for: Clinical Factors Associated with SFTS Diagnosis and Severity in Cats
Source: Viruses. 2024 May 29;16(6):874. doi: 10.3390/v16060874 (PMC11209305; doi:10.3390/v16060874)
Supplement: Supplementary file 1 [file viruses-16-00874-s001.zip › Table S2.pdf]

Table S2. Comparison of the clinical characteristics of SFTSV-positive and SFTSV-negative cases, related to Figure1.

			SFTSV-positive		SFTSV-negative		
**Clinical parameter**	**Reference**	**Total**	**Median (IQR)**	**N**	**Median (IQR)**	**N**	**P-value**
Age (yrs)	-	175	4 (2-8)	105	3 (1-7)	70	0.220
Body weight (kg)	-	181	3.7 (3-4.5)	105	4.0 (3.3-4.7)	76	0.046*
Body temperature (°C)	38.0-39.0	170	39.5 (38.5-40.1)	100	39.5 (38.7-40.1)	70	0.963
RBC ($\times10^4/\mu L$)	500-1000	164	693 (5375-8725)	95	803 (699-886)	69	0.004**
WBC ($/\mu L$)	5500-19500	169	7250 (3610-14278)	98	4250 (2235-6650)	71	<0.001***
PLT ($\times10^3/\mu L$)	300-800	159	80 (44-150)	93	58 (35-90)	66	0.014*
ALT (IU/L)	6-83	162	68 (41-137)	94	64 (49-101)	68	0.791
AST (IU/L)	26-43	143	59 (32-159)	82	79 (48-153)	61	0.034*
CPK (IU/L)	7.2-28.2	113	275 (192-577)	67	245 (172-480)	46	0.567
TBil (mg/dL)	0.15-0.5	154	2 (1.0-3.8)	89	4.2 (1.2-7.3)	65	0.005**

Each variable was compared in the SFTSV-positive and SFTSV-negative cases using the Wilcoxon rank-sum test, and statistical significance is shown: *$p < 0.05$, **$p < 0.01$, ***$p < 0.001$. IQR, interquartile range; N, number of cases; Reference, reference range of each variable; SFTSV, severe fever with thrombocytopenia syndrome virus.
