# Supplementary material for: Clinical Factors Associated with SFTS Diagnosis and Severity in Cats
Source: Viruses. 2024 May 29;16(6):874. doi: 10.3390/v16060874 (PMC11209305; doi:10.3390/v16060874)
Supplement: Supplementary file 1 [file viruses-16-00874-s001.zip › Table S3.pdf]

Table S3. Comparison of the clinical characteristics of surviving and fatal SFTSV-negative cases, related to Figure 2.

		Survival		Dead		
**Clinical parameter**	**Total**	**Median (IQR)**	**N**	**Median (IQR)**	**N**	**P-value**
Age (yrs)	55	4.0 (2.0-6.0)	33	5.5 (2.3-12.8)	22	0.095
Body weight (kg)	55	4.0 (3.1-4.8)	33	3.3 (2.8-5.0)	22	0.130
Body temperature (°C)	50	39.6 (38.5-40.2)	30	38.3 (36.6-39.5)	20	0.009**
RBC ($\times 10^4/\mu L$)	50	825 (642-939)	30	647 (476-697)	20	0.027*
WBC ($/\mu L$)	50	9800 (6733-14275)	30	5695 (950-16998)	20	0.281
PLT ($\times 10^3/\mu L$)	48	102 (60-139)	30	73 (37-103)	18	0.157
ALT (IU/L)	49	67 (49-116)	29	101 (40-231)	20	0.369
AST (IU/L)	44	52 (37-80)	28	150 (64-259)	16	0.051
CPK (IU/L)	41	227 (174-404)	23	377 (278-562)	18	0.031*
TBil (mg/dL)	44	1.7 (1.0-2.7)	26	2.05 (1.6-6.1)	18	0.177

Each variable was compared in the surviving and fatal SFTSV-negative cases using the Wilcoxon rank-sum test, and the statistical significance is shown: *$p < 0.05$, **$p < 0.01$, ***$p < 0.001$. IQR, interquartile range; N, number of cases; SFTSV, severe fever with thrombocytopenia syndrome virus.
