# Supplementary material for: Clinical Factors Associated with SFTS Diagnosis and Severity in Cats
Source: Viruses. 2024 May 29;16(6):874. doi: 10.3390/v16060874 (PMC11209305; doi:10.3390/v16060874)
Supplement: Supplementary file 1 [file viruses-16-00874-s001.zip › Table S4.pdf]

Table S4. Comparison of the clinical characteristics of surviving and fatal SFTSV-positive cases, related to Figure 2.

		Survival		Dead		
**Clinical parameter**	**Total**	**Median (IQR)**	**N**	**Median (IQR)**	**N**	**P-value**
Age (yrs)	56	3.0 (1.0-5.0)	24	3.0 (1.0-8.0)	32	0.839
Body weight (kg)	60	4.0 (3.5-4.3)	25	3.9 (3.3-4.5)	35	0.826
Body temperature (°C)	54	39.4 (39.1-39.9)	21	39.5 (38.5-40.1)	33	0.708
RBC ($\times 10^4/\mu L$)	53	808 (755-889)	22	788 (710-885)	31	0.697
WBC (/μL)	55	4600 (2650-6450)	22	3300 (1400-4660)	33	0.058
PLT ($\times 10^3/\mu L$)	50	64 (37-99)	19	51 (28-79)	31	0.582
ALT (IU/L)	52	55 (42-78)	21	81 (52-119)	31	0.021*
AST (IU/L)	46	69 (49-86)	20	150 (58-350)	26	0.008**
CPK (IU/L)	33	232 (142-377)	15	360 (192-831)	18	0.095
TBil (mg/dL)	49	3.4 (2.6-5.2)	21	4.8 (1.4-7.9)	28	0.242

Each variable was compared in the surviving and fatal SFTSV-positive cases using Wilcoxon rank-sum test, and the statistical significance is shown;*p < 0.05, **p < 0.01, ***p < 0.001. IQR, interquartile range ; N, number of cases; SFTSV, severe fever with thrombocytopenia syndrome virus.
