# Supplementary material for: Clinical Factors Associated with SFTS Diagnosis and Severity in Cats
Source: Viruses. 2024 May 29;16(6):874. doi: 10.3390/v16060874 (PMC11209305; doi:10.3390/v16060874)
Supplement: Supplementary file 1 [file viruses-16-00874-s001.zip › Table S5.pdf]

Table S5. Comparison of the RNA levels of surviving and fatal SFTSV-positive cases, related to Figure 3.

	Survival		Dead		
**Specimen**	**Median (IQR)**	**N**	**Median (IQR)**	**N**	**P-value**
Serum	221 (141-2409)	5	347922 (128706-239779062)	14	0.019*
Eye swabs	14853 (13381-16325)	2	49 (34-126)	5	0.381
Oral swabs	4112 (173-20824)	6	419 (70-1267)	11	0.525
Anal swabs	176 (103-3122)	5	1613 (77-31016)	11	0.510

The RNA levels were compered in the surviving and fatal SFTSV-positive cases using the Wilcoxon rank-sum test. The unit of RNA levels is copies/5-μL. The statistical significance is shown;*p < 0.05, **p < 0.01, ***p < 0.001. IQR, interquartile range ; N, number of cases; SFTSV, severe fever with thrombocytopenia syndrome virus.
